# Monthly Summary

**Published:** 1891-01

**Authors:** 


### Monthly Summary.

ART AND DENTISTRY.—A good dentist should, indeed, be a man of great refinement, of artistic conception, with a true sense of the proportion of things, and of the harmony of colors. We have only to look at the teeth people often wear to notice that this is not very often the case. It must be remembered that in nature there is a great beauty in the irregularities, in what is often called the ugliness of shape and color. Because an even row of very white teeth is the ideal, this does not prove that such teeth suit everybody. What can be more ghastly than an old, decrepit personage, with a bad complexion, who sports a double row of splendid white teeth? What is more ridiculous than one white, spotless artificial tooth standing in the midst of yellow and partially decayed real teeth? Or, again, what a lop-sided effect is produced if teeth on one side of the mouth grow irregularly, while, on the other side, artificial teeth have been fixed up in regimental order. Yet how few people are there who, having artificial teeth, have the good sense to ask that those teeth should be just as imperfect in shape, position and color as the real teeth were they are destined to replace?

If we have not ideal teeth, the probabilities are that there are many other things in feature and complexion which also are far from being ideal. And the introduction of one or more ideal teeth, where the surroundings are anything but ideal, is no improvement. It creates a discordant note, destroys the harmony which prevails even in ugliness, and renders that ugliness more evident and more unpleasant. But it requires a high conception of true art to thoroughly appreciate these principles and apply them successfully in practice. It is, therefore, not surprising to find that distinguished dentists are the constant and appreciated friends of men of art and of letters.—*Dental Advertiser.*

---

LATER OPINIONS ON THE VALUE OF KOCH'S DISCOVERY.—Professor Virchow defends Professor Koch against the charge of prematurely publishing his discovery. Professor Koch, he says, only consented to the disclosures already made at the request of Minister von Gossler and several of his medical colleagues, Doctors Virchow, Levy and Bergmann. Everyone in Professor Koch's confidence supports his protest against the sensational expectations regarding the results of the remedy. Professor Stellwag, of the Vienna University, addressing the students, advised them to be cautious in expectance, believing only what Professor Koch has directly stated. "So far," added Professor Stellwag, "the possibility of the cure of lupus alone has been proved, while it has not been scientifically established that lupus arises from the same bacillus that is associated with lung tubercles. Dr. Ullmann writes: "It will take fully a year of frequent injections, besides treatment under right sanitary conditions, to enable one to form a reliable opinion as to the curability of consumption, either in advanced or in early stages." Doctor Ullmann worked for several months in Professor Koch's laboratory. He believes the remedy promises good results in cases of external tuberculosis, although, he says, relapses must be guarded against. Doctor Surycki, reporting to the Medical Society of Cracow, which sent him to Berlin to investigate, declares that even the cure of external tuberculosis by the new process is uncertain, while he sees no grounds for believing that it will cure con sumption in any stage. Doctor Kraus, of Vienna, affirms the benefit of the remedy for tuberculosis of the bones, skin, and joints, but does not believe that it will ever heal lung tubercles. Doctor Kraus was in attendance in Berlin for several weeks, testing the experiments. Professors Fraenzel and Runkuritz, in their latest report, confirm the opinion that the injections do not materially check advanced phthisis. They do, however, arrest early phthisis, but the bacilli may revive and reinfect the tissues. The opinions of a number of other experts—German, Austrian, and English—all of the same tenor, are becoming known, and tone down the excited public expectations.—*Med. Record.*

---

RETAINING POINTS.—Nowadays it is the fashion to declaim against retaining points for cavities in which gold is to be inserted. Never was there a greater fallacy. Take the simplest contour filling, which may be one in an incisor tooth with a living pulp, and it goes without saying that the first pieces of gold should be securely fastened to a given point or points to insure non-tipping. Be the cavity ever so well prepared for retention of gold, a point or two in the thicker portions of the labial or palatal wall and one near the cutting edge, or at least a properly shaped groove, is indispensable for the prevention of leakage into the cavity. Other cavities in living teeth call for the use of retaining points—not in the direction of the pulp—for the pinning of gold to the abraded ends of the teeth. Many times pulpless teeth are rendered more useful by the drilling of retaining pits in the proper direction, saving strength of tooth structure and avoiding the loss of profile walls whose cutting away would disfigure the countenance, by restoration with gold. Operations on the teeth with gold under the most favorable circumstances are transitory instead of permanent, and it were death to estheticism to avoid the use of pits or points as an aid to perfection of mechanical impaction of gold to prevent unsightly discoloration, in order to say that all cavities should be prepared in a manner that renders unnecessary the boring or drilling of pits, or points which will permit of the pinning of gold to a cavity. Selah!—*Dental Review.*

